# The Effect of Presenteeism on Productivity Loss in Nurses: The Mediation of Health and the Moderation of General Self-Efficacy

**DOI:** 10.3389/fpsyg.2019.01745

**Published:** 2019-07-31

**Authors:** Yongxin Li, Jihao Zhang, Shengnan Wang, Shujie Guo

**Affiliations:** ^1^Institute of Psychology and Behavior, Henan University, Kaifeng, China; ^2^Nursing Department, Henan Province People’s Hospital, Zhengzhou, China

**Keywords:** presenteeism, productivity loss, health, general self-efficacy, nurse, China

## Abstract

**Background:** Seventy-four percent of Chinese employees have experienced working with illness, but limited number of researchers have paid attention on this phenomenon. Most of the previous research on presenteeism has almost exclusively focused on North America and Europe and have gone to the financial emphasis. The current researches have two shortages, which are laying in the consensus on the definition and measurement of presenteeism, as well as the mechanism of presenteeism and its outcomes have set barriers for scholars to generate deeper understanding of the behavior. The aim of the present study was to explore the current situation of presenteeism among Chinese nurses and the mediating effect of health and the moderating effect of general self-efficacy between presenteeism and productivity loss.

**Methods:** Data were collected from a sample of 340 female nurses from a 3A-graded general hospital in Henan Province, China by using the Sickness Presenteeism Questionnaire (SPQ), the Stanford Presenteeism Scale (SPS-6), the 12-item General Health Questionnaire (GHQ-12), and the General Self-Efficacy Scale (GSES).

**Results:** The results indicated that the mean of SPQ was 3.2 ± 0.7 in this sample, and there were significant differences in age and marital status in SPQ scores. Presenteeism was significantly associated with health and productivity loss, and health was significantly associated with productivity loss, and general self-efficacy was negatively associated with productivity loss. A bootstrap test showed that health fully mediated the relationship between presenteeism and productivity loss in nurses. Hierarchical regression analysis confirmed the moderating role of general self-efficacy between presenteeism and productivity loss.

**Conclusions:** Presenteeism can significantly predict productivity loss in nurses, and hospital management can improve the physical and mental health of nurses and enhance their self-efficacy level to reduce the negative impact of presenteeism on productivity loss.

## Introduction

Presenteeism, which is defined as “ill and still work” (Aronsson et al., 2000), has become a common phenomenon in the workplace. About 88% of employees and 85% of healthcare providers have worked while being sick (McKevitt et al., 1997). In 2005, more than 65.6% of the Canadian government staff reported working while ill, and the average amount of time was 11.9 days per year when they felt sick but had to work (Biron et al., 2006). Likewise, the nursing professions are prone to presenteeism due to heavy workloads, shift work, and irreplaceable duties (Bergstrom et al., 2009a,b). Presenteeism not only negatively effects on the quality of the nurses’ care, job satisfaction, and job preference, but it may also lead to direct and indirect loss to organizations (Zhang et al., 2017). Some scholars have estimated that the average annual loss from nurses’ presenteeism in North Carolina is around 2–13 billion dollars (Letvak et al., 2012). Therefore, the phenomenon of the presenteeism of nurses has attracted more and more attention of scholars in the mental health, nursing management, public hygiene, and occupational health domains in recent years (Virtanen et al., 2003; Pilette, 2005).

During the last 20 years, there has been great progress into the study of nurses’ presenteeism. Several surveys have been conducted on the occurrence percentage of presenteeism in North America and Europe (Lin and Lu, 2013). Some predicting variables of presenteeism, such as depression (Koopman et al., 2002), work stress (Elstad and Vabo, 2008), job demand (Demerouti et al., 2009), and a sense of being irreplaceable (Bergstrom et al., 2009a,b), have been proposed and supported by empirical research. Presenteeism has also been found to be related to a series of attitudinal and behavioral variables, such as individual health (Gosselin et al., 2013), quality of care (Letvak et al., 2012), job satisfaction (Baker-McClearn et al., 2010), and job burnout (Ferreira and Martinez, 2012).

However, studies on presenteeism have two limitations. The first is the lack of consensus on the definition and measurement of presenteeism. Most European scholars focus on “ill” and “work” when discussing the definition of presenteeism, and the majority of them prefer to measure its occurrence (Aronsson et al., 2000). While American scholars tend to define presenteeism according to the productivity loss that is caused by employees’ “ill and still work” and have highlighted the calculation of economic cost (Turpin et al., 2004). The different approach to the concept may hinder communication between researchers and induce confusion on how to prevent presenteeism in practice. Johns (2010, 2012) claimed that presenteeism could be caused by job insecurity, work stress, and economic needs. But unlike absenteeism, presenteeism sometimes may foster productivity rise rather than loss. Thus, the definition of presenteeism should focus on the behavior itself rather than its antecedents and outcomes. Hence, it is necessary to clarify the concept connotation and make a strict distinction between the behavior and its outcomes (e.g., productivity loss), measure its existence situation rather than its outcomes, and establish links between them.

Since previous studies mainly focused on investigating presenteeism’s influential factors (Aronsson et al., 2000; Johansson and Lundberg, 2004; Aronsson and Gustafsson, 2005; Hansen and Andersen, 2008; Johns, 2011) rather than its negative outcomes (Bergstrom et al., 2009a,b; Gustafsson and Marklund, 2011), the second limitation is the lack of inquiry into the mechanism of presenteeism and its outcomes. Although scholars have proposed integrated frameworks of presenteeism (Johns, 2010), there are few empirical studies exploring the acting mechanism of the behavior’s antecedents, nature, and outcomes within a whole framework. Consequently, this impedes researchers in their ability to acquire knowledge about the motivation and outcomes of presenteeism. Therefore, subsequent studies on presenteeism should start with the relationships between variables and explore the related boundary conditions.

**Figure 1:**
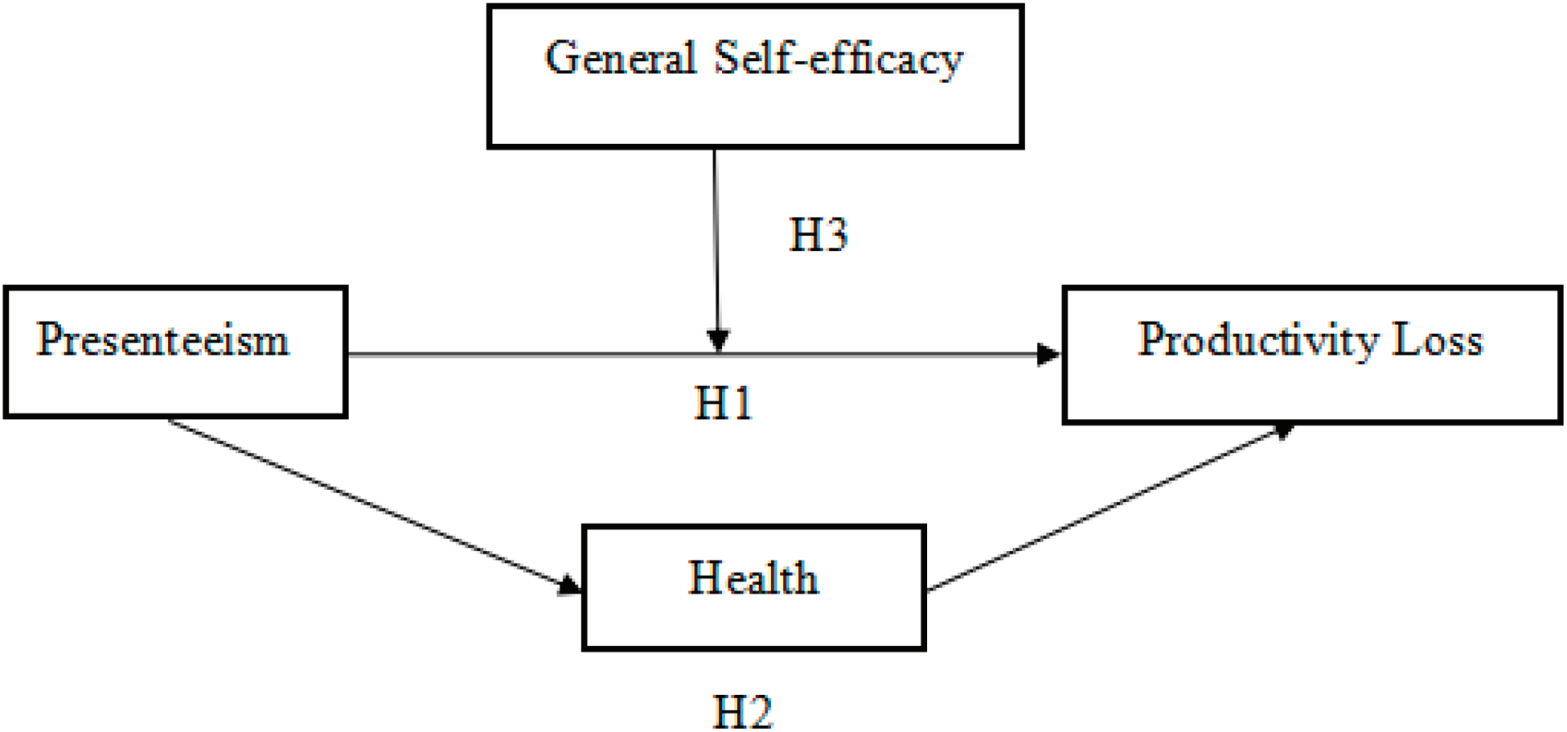
The hypothesized model.

Meanwhile, one investigation reported that 74% of Chinese employees have experienced working with illness in 2010 (Sun and Zhang, 2015), and the widespread culture of working overtime in Chinese enterprises caused employees to exhibit more presenteeism (Lin and Lu, 2013). The causes of this phenomenon may be attributed to the global economic recession and the rising numbers of unemployment in recent years, which to a certain extent increased the demands for excessive overtime work and the accountability system in organizations. Yet, most research has been conducted in Europe and North America, while Chinese scholars have paid less attention to this issue (Zhang and Li, 2016). The limited amount of research that has been done in China has not been empirical but mainly focused on the introduction and review of presenteeism, rarely exploring the acting mechanism between the relevant variables. This research aims to explore the relationship between presenteeism and productivity loss among Chinese nurses by analyzing the mediation role of health and the moderation role of general self-efficacy. Additionally, by investigating the current situation of presenteeism in nurses, the present study could provide empirical evidence for medical and health management to improve nurses’ health and their work efficiency by implementing interventions for those working with illness, thus enabling them to offer better services to their patients. The hypothesized model was shown in Figure 1.

## Theory and Hypotheses

### Presenteeism and Health-Related Productivity Loss

Health-related productivity loss has been defined as a decline in an individual’s productivity due to presenteeism and absenteeism for illness (Koopmanschap et al., 2005; Beaton et al., 2009). Previous studies have paid more attention to the negative effects of illness absenteeism on health-related productivity but less on the great loss that results from presenteeism (Goetzel et al., 2004). However, evidence has revealed that the loss of productivity that presenteeism brings to organizations is far greater than that of sick leave (Weaver, 2010). As the incidence of presenteeism in the nursing professions is significantly higher than that of other professions (Aronsson et al., 2000), its negative consequences to nurses should not be ignored. On the one hand, the direct result of medical and nursing staff working while ill is the subsequent inefficiency of work and the increase in errors, which indirectly leads to productivity loss. On the other hand, due to the particularity of medical and nursing work, presenteeism may adversely affect the health security of patients. Letvak et al. (2012) reported that nurses’ presenteeism often leads to an increase in patients’ falls and medication errors and to a reduction in service quality, and the annual per capita economic loss caused by presenteeism among nurses in North Carolina was reported to be between $1,346 and $9,000. Rantanen and Tuominen (2011) found that 37.4% of the nurses had been working with presenteeism during the past 4 weeks and, compared with the normal state, the loss of the nurses’ ability to work during the duty period was up to 45.4%. Based on the evidence mentioned above, we therefore hypothesize the following:

*Hypothesis 1:* Nurses’ presenteeism is significantly positive related to health-related productivity loss.

### Mediation Effects of Health

Health is a state of dynamic balance, involving a balanced input and output of energy and matter. According to the conservation of resources theory, as the total amount of an individual’s resources, such as time and energy, is limited, an individual will automatically consume subordinate resources to maintain its prior resources (Hobfoll, 2011). At the same time, the effort-recovery theory holds that employees need enough resources to recover physically and mentally after work. If the resource is deficient and the recovery is insufficient, the nervous system will remain active, and the person may not be able to recover to a self-balanced state (Meijman and Mulder, 1998). Also, employees in a suboptimal health condition will have to make extra effort and consume more resources to cope with the subsequent work requirements, which may lead to further long-term fatigue (Sonnentag et al., 2012). However, according to the effort-recovery theory, employees need resource restoration when they are ill. These resources may include time to rest and the ability to be out of work, but their attendance at work deprives these individuals of the opportunity to recover from stress and disease, resulting in depleted resources, fatigue accumulation, and stress symptoms, which is likely to cause further deterioration of the individual’s health, as it could bring about serious physiological and psychological consequences.

According to the world health organization’s definition of “health,” it includes not only physical health but also mental health (Callahan, 1973). Studies have also found that presenteeism has a direct negative effect on nurses’ health (Karimi et al., 2014; Lu et al., 2014). Kivimaki et al. (2005) found that employees in the high-level presenteeism group were twice more likely to suffer from heart disease than those in the low-level group. Meanwhile, studies have shown that there is a significant negative correlation between presenteeism and the mental health level of nurses (D’Errico et al., 2013). Demerouti et al. (2009) conducted a longitudinal study based on the nurses group and found that presenteeism has not only directly led to individual fatigue, tension, anxiety, and depersonalization but also had a continuous negative effect, which would further damage individual physical and mental health. Since nurses perform the majority of the nursing work in hospital, the health status of nurses not only directly affects the efficiency of the nurses’ work, but it also affects the rehabilitation, treatment effect, and the harmony of the relationship between doctors and patients. Besides that, a great deal of evidence has proven that serious consequences such as a quality decline, efficiency slide, and productivity sabotage, are very likely to occur when nurses are working under unhealthy conditions (Prasad et al., 2004). More precisely, the poorer the physical and mental health of employees, the greater the proportion of their productivity that would be impaired (Zhao et al., 2011). In view of these findings regarding the literature, we hypothesize the following:

*Hypothesis 2:* Health plays an intermediary role between nurse presenteeism and productivity loss.

### Moderation Effects of General Self-Efficacy

Bandura’s social cognitive theory suggests that self-efficacy has a large influence on a person’s success in activities and tasks and whether the engaged process is favorable or not since the individual’s self-efficacy restricts his or her level of motivation, actions, and psychological state (Bandura, 1997). Johns (2010) have proposed an integrated framework of presenteeism within which personality was an important variable. Also, the findings of Dew et al. (2005) suggested that self-efficacy, which is a crucial component of personality traits, may operate as a moderator in the relationship between presenteeism and its outcome variables. However, there were few empirical researches concerned about the role of self-efficacy to date. As a response to the research of Dew et al. (2005), self-efficacy has been introduced as a moderator basing on the social cognitive theory (Bandura, 1997) and Johns’ integrated framework (Johns, 2010) to examine the acting mechanism of presenteeism on its outcomes. However, compared to Bandura’s concept of self-efficacy, the following concept of general self-efficacy is a comparatively stable personality traits and a core variable in the individual’s self-belief system, which can be regarded as the individual’s overall confidence in dealing with the challenges of various situations or the ability to face burgeoning issues. It can predict individuals’ behaviors in different situations (Schwarzer and Aristi, 1997). Individuals with high self-efficacy usually possess a positive self-image and a high level of confidence, which empower them to cope with the various stresses in their professions effectively and, subsequently, to maintain good health and a high level of work performance (Bandura, 1997). Lu et al. (2005) found that self-efficacy regulates the negative impact of stress on individual health. Meanwhile, Feng et al. (2008) examined the moderation effect of general self-efficacy on the relationship between job insecurity and individual job performance. Therefore, we propose the following hypothesis:

*Hypothesis 3:* General self-efficacy moderates the positive relationship between the nurses’ presenteeism and productivity loss, and for the nurses with a high level of general self-efficacy, the relationship will be weakened.

## Materials and Methods

### Participants and Procedure

In the present study, we distributed questionnaires to 370 in-service nurses from a 3A-graded general hospital at Henan Province, China and received 340 valid responses. All the participants are female. Because in China, women occupy the majority of the profession. In the hospital we surveyed, there were about 3,000 nurses, fewer than 50 of them are male nurses. In this survey, we only received seven responses from the male nurses, the male sample was too small to be included in the statistical analysis. Moreover, all the participants were informed of the research purpose and the confidential principles before the survey, and then, we conducted anonymous surveys in each department as a unit with consent from the participants. The average age of the participants was 28 ± 4.63 years old; 195 (57.4%) of the participants were married, while 145 (42.6%) were not; 261 participants (76.8%) had college degrees, while 79 (23.2%) did not. About 195 participants (57.4%) had a work tenure of 5 years or less, 85 participants (25%) had 6–10 years of tenure, and 60 (17.6%) participants had 11–15 years tenure.

### Measures

#### Presenteeism

To measure nurses’ presenteeism, Lu et al.’s (2013) unidimensional two-item Sickness Presenteeism Questionnaire (SPQ) was adopted. The two items on the questionnaire are: “Although you feel sick, you still force yourself to go to work” and “Although you have physical symptoms such as headache or backache, you still force yourself to go to work.” Nurses were asked to indicate the degree to which each statement described their performance of these behaviors during the last 6 months on a four-point Likert scale (1 = never, 2 = once, 3 = 2–5 times; 4 = more than 5 times). The higher the score, the higher the frequency of presenteeism. In this study, the Cronbach’s alpha was 0.84.

#### Productivity Loss

Health-related productivity loss was measured *via* the six-item Chinese version of the Stanford Presenteeism Scale (Zhao et al., 2010), which was originally compiled by Koopman et al. (2002) and widely used to assess the impact of health problems on individual’s productivity (Koopman et al., 2002). It contains six items, including two dimensions of completing work (four items, e.g., “Despite having my health problem, I was able to finish hard tasks in my work.” requiring reverse scoring) and avoiding distraction (two items, e.g., “My health problem distracted me from taking pleasure in my work.”). All the items were scored on a five-point Likert scale, ranging from 1 (completely disagree) to 5 (totally agree), and the score range was 6–30. The higher the score from SPS-6, the greater the loss of health-related productivity caused by the presenteeism of the participants. In this study, the Cronbach’s alpha was 0.72.

#### General Health

Goldberg et al.’s (1997) 12-item General Health Questionnaire (GHQ-12) was employed to measure participants’ perceptions regarding their health conditions. The questionnaire concerns on two areas of normal dysfunction and recent-appeared distressing situations, to assess individual current state and inquiry is there any differences from usual state (Goldberg, 1972; Fryers et al., 2004). It is composed of six positive items (sample items are “Have you been able to concentrate on whatever you doing” etc. reverse scoring) and six negative items (sample items are “Have you lost much sleep over worry” etc.). Likewise, all the items are scored on a four-point Likert scale, ranging from 1 (never) to 4 (usually). Standard Likert summation ranges from 12 to 48. Similarly, the higher the score reflecting the lower health level of the participants (Gnambs and Staufenbiel, 2018). The questionnaire has been successfully conducted in Chinese samples and already been proven to obtain good psychometric properties (Cai et al., 2016). In this study, the Cronbach’s alpha was 0.77.

#### General Self-Efficacy

To measure nurses’ general self-efficacy, the revised Chinese version of the general Self-Efficacy Scale (GSES) (Wang et al., 2001), which was developed by Schwarzer and Aristi (1997) and widely used internationally, was adopted. The scale includes 10 items, such as “I am confident that I can deal with anything unexpected.” All the items are scored on a four-point Likert scale, ranging from 1 (completely inconsistent) to 4 (completely consistent). The theoretical score range is from 10 to 40. Higher scores indicate that the participants have a higher level of general self-efficacy. In the present study, the Cronbach’s alpha for this questionnaire was 0.90.

### Data Analysis

SPSS 22.0 and a bootstrap technique were applied to analyze the collected data, and the specific data analysis consisted of the following steps. First, a descriptive analysis of the SPQ scores from participants was used. To be precise, an independent sample *t*-test and a variance analysis were used to compare the scores from different groups of nurses with a variety of ages, marital statuses, educational levels, and tenure. Second, a Pearson correlation analysis was manipulated to test the correlations between variables. Third, a bootstrap technique was adopted to examine the mediation of health on presenteeism and productivity loss. Finally, a hierarchical regression analysis was used to examine the moderating effects of general self-efficacy between presenteeism and productivity loss, and a simple slope test (Aiken and West, 1991) was used to verify the moderating effect.

## Results

### Nurses’ Sickness Presenteeism Questionnaire Scores and the Differences in Demography Characteristics

Tables 1, 2 present the descriptive statistics of the nurses’ scores from the SPQ. The overall mean score of the SPQ is 3.2 ± 0.7, which is notably higher than the theoretical mean of the four-point Likert questionnaire. Specifically, 83.5% (284) of the participants rated 3 or 4 for item 1, while 86.8% (294) rated 3 and 4 for item 2. The demographic differences of the nurses’ SPQ scores are listed in Table 2. As shown in the table, presenteeism had a significantly positive relationship to the nurses’ marital status; married nurses preferred to work while ill more so than unmarried nurses (*t* = −2.79, *p* < 0.01). Also, nurses of different ages had significant differences in SPQ scores (*F* = 3.15, *p* < 0.05), and further *post hoc* analysis discovered that the presenteeism of nurses at the age of 31 and above was significantly more than that of the nurses 25 years old and younger.

**Table 1:** Means of the SPQ (%).

Items	1 (Never)	2 (Once)	3 (2–5 times)	4 (>5 times)
1. Although you feel sick, you still force yourself to go to work.	13(3.8)	43(12.6)	152(44.7)	132(38.8)
2. Although you have physical symptoms such as headache or backache, you still force yourself to go to work.	9(2.6)	36(10.6)	139(40.9)	156(45.9)

*SPQ, Sickness Presenteeism Questionnaire*.

**Table 2:** Descriptives and correlations among the demographic characteristics and SPQ scores.

Variables	Categories	Case	*x* ± *s*	*t*/*F*	*p*
Age/year	≤25	107	3.11 ± 0.79	3.15	0.038
	26–30	163	3.27 ± 0.69		
	≥31	70	3.38 ± 0.69		
Marital status	Unmarried	145	3.12 ± 0.71	−2.79	0.006
	Married	195	3.34 ± 0.72		
Education level	College and below	79	3.14 ± 0.73	−1.46	0.147
	Bachelor and above	261	3.27 ± 0.72		
Tenure	≤5	195	3.22 ± 0.74	0.61	0.543
	6–10	85	3.24 ± 0.71		
	≥11	60	3.33 ± 0.69		

### Variables Correlations

As shown in Table 3, presenteeism had a significantly positive relationship to health (*r* = 0.20, *p* < 0.01) and health-related productivity loss (*r* = 0.17, *p* < 0.01). Health was positively associated with health-related productivity loss prominently (*r* = 0.32, *p* < 0.01), whereas it was negatively related to general self-efficacy (*r* = −0.26, *p* < 0.01). While general self-efficacy was negatively associated with health-related productivity loss (*r* = −0.17, *p* < 0.01). Hence, Hypothesis 1 was supported.

**Table 3:** Variables correlations.

	*M*	SD	1	2	3	4	5	6	7	8
1. Age	0.89	0.72	1.00							
2. Marital status	0.58	0.50	0.64^**^	1.00						
3. Educational level	0.77	0.42	0.39^**^	0.27^**^	1.00					
4. Tenure	0.60	0.77	0.76^**^	0.50^**^	0.26^**^	1.00				
5. SPQ	3.24	0.72	0.13^*^	0.15^**^	0.08	0.06	1.00			
6. GHQ-12	2.24	0.41	0.09	0.09	0.02	0.01	0.20^**^	1.00		
7. GSSE	2.62	0.54	0.08	0.05	−0.04	0.13^*^	−0.05	−0.26^**^	1.00	
8. SPS-6	2.70	0.80	0.05	0.14^**^	0.07	0.08	0.17^**^	0.32^**^	−0.17^**^	1.00

*N = 340; age: 0 = ≤25; 1 = 26–30; 2 = ≥31. Marital status: 0 = unmarried, 1 = married. Educational level: 0 = College and below, 1 = Bachelor and above. Tenure: 0 = ≤5; 1 = 6–10; 2 = ≥11. SPQ, Sickness Presenteeism Questionnaire; GHQ-12, General Health Questionnaire; GSES, General Self-Efficacy Scale; SPS-6, Stanford Presenteeism Scale*.

* *p < 0.05*;

** *p < 0.01*.

### Mediation Effects of Health on Nurses’ Presenteeism Behavior and Health-Related Productivity Loss

The bootstrapping analysis with 5,000 iterations has been adopted to examine the mediating effect of health on nurses’ presenteeism behavior and health-related productivity loss. The pluggable unit of PROCESS in SPSS, which is developed by Hayes (2014), was applied to analyze the mediation effect of health. Step one, put control variables of age, marital status, educational level and tenure into the covariate box. Step two, place the independent variable of the nurses’ presenteeism behavior, the mediating variable of health, and the outcome variable of health-related productivity in the corresponding box. Step three, set the model as a four-factor model, and the sample size was set to be 5,000, and the confidence interval was set to 95% to get the analysis results. If 95% CI does not include zero, the mediating effect is statistically significant. The results revealed a significant mediation effect of health between nurses’ presenteeism and productivity loss, as the 95% bias-corrected confidence interval for the indirect effect of health excluded zero (CI = [0.02, 0.11]), and the mediator effect was 0.06. In contrast, by controlling the mediation variable of health to investigate the direct effect of the nurses’ presenteeism on productivity loss, the direct effect was not significant (95% CI = [−0.07, 0.11], *t* = 1.85, *p* > 0.05). Therefore, it could be concluded that health played a complete mediation role between nurses’ presenteeism and productivity loss, and the indirect effect accounted for 36% of the total effect. Hence, Hypothesis 2 was supported.

### Moderation Effects of General Self-Efficacy Between Nurses’ Presenteeism and Health-Related Productivity Loss

The score of presenteeism and general self-efficacy was centralized to reduce the collinearity, and the product of the two was calculated as interaction item. As shown in Table 4, the main effect of presenteeism (*β* = 0.15, *p* < 0.01), general self-efficacy (*β* = −0.18, *p* < 0.01) on productivity loss is significant, and the interaction of the two also has a significant predictive effect on productivity loss (*β* = −0.18, *p* < 0.01). Moreover, when we introduced the product of the two variables into the regression equation, the ΔR^2^ of the latter two regression equations equaled 0.03, *p* < 0.01, which reached to a significant level. The results provided evidence that general self-efficacy has a significant moderation effect on the relationship of nurses’ presenteeism and productivity loss. Thus, the third hypothesis was confirmed.

**Table 4:** Results of the HRA of the moderation effect of general self-efficacy.

Variables	Productivity loss		
	Step 1	Step 2	Step 3
Control variables			
Age	−0.17	−0.19^*^	−0.18
Marital status	0.19^**^	0.17^*^	0.17^*^
Educational level	0.06	0.04	0.04
Tenure	0.10	0.14	0.14
Main effect			
Presenteeism		0.15^**^	0.18^**^
General self-efficacy		−0.18^**^	−0.19^***^
Moderation effect			
Presenteeism × general self-efficacy			−0.18^**^
*F*	2.69^*^	5.17^***^	6.19^***^
Δ*R*^2^	0.03^*^	0.05^***^	0.03^**^

*N = 340, Δ*R*^2^ refers to the added value of the proportion of the variation in the dependent variable that is explained by the regression equation as the new independent variable continues to enter the model*.

* *p < 0.05*;

** *p < 0.01*;

*** *p < 0.001*.

**Figure 2:**
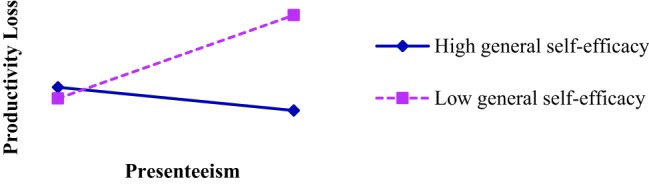
Simple slope analysis.

Furthermore, a simple slope test was conducted to examine the moderating trend of general self-efficacy on the relationship between presenteeism and productivity loss by examining the plus and minus standard deviation from the mean of the two variables (see Figure 2). As indicated in Figure 2, under the condition of low general self-efficacy, the nurses’ presenteeism had a significant predictive effect on productivity loss (*β* = 0.46, *p* < 0.01), which means the nurses with low general self-efficacy tend toward presenteeism and suffer greater impairment of their productivity. While in the high general self-efficacy group, the predictive effect of nurses’ presenteeism on productivity loss was not significant (*β* = −0.02, *p* > 0.05).

## Discussion

### General Discussion

In this research, a detailed investigation of a presenteeism situation has been carried out, in which selected in-service nurses from a 3A-graded general hospital at Henan province, China were used as samples. The results of the study have proven the mediation effect of health and the moderation effect of general self-efficacy on the relationship between presenteeism and health-related productivity loss. The concrete analysis follows.

First, the mean score of the nurses’ presenteeism in this study was 3.2 ± 0.7, which indicates that in the sample of this survey, the frequency of the nurses’ presenteeism was notably high. This result is consistent with the results of previous studies (Elstad and Vabo, 2008; Demerouti et al., 2009). Moreover, the results illustrated that there were significant differences in participants’ demographic characteristics, such as age and marital status, but there was no significant difference between educational level and tenure. The results illustrated that the frequency of presenteeism was in accordance with the increase of age among nurses; the presenteeism of nurses age 31 and older was significantly more than that of nurses 25 years old and younger, which is noteworthy as well. Indeed, this may be attributed to the fact that the older nurses were less confident in seeking new job opportunities and their pursuit of a stable life. Most of the older nurses might have been afraid that frequent absenteeism behavior might cause them to lose their jobs; thus, they often chose to force themselves to work even when they were ill (Johns, 2012). In spite of that, the older employees seemed to have formed a relatively negative moral perspective of the absence of work due to their long tenure of working experience; therefore, for reasons of organizational norms, they participated in presenteeism to avoid absenteeism (Bierla et al., 2013). Furthermore, to explain the higher scores of presenteeism of the married nurses than that of the unmarried, it may be that the married ones not only bear the heavy burden of family affairs and responsibilities, such as taking care of children, but they also have higher demands for financial security to support their families (Sasmita and Sneha, 2013).

Second, the results confirmed the complete mediation effect of health on the relationship of presenteeism and health-related productivity loss in nurses. The conservation of resources theory (Hobfoll, 2011) may provide explanations for this conclusion. To complete their job tasks, nurses who work with illness often need to consume extra physical, psychological and even emotional resources. However, these extra efforts sometimes fail to guarantee desirable consequences of the task; in other words, no matter whether the outcome of the task is positive or not, a large amount of resources will still be consumed. As the total amount of an individual’s resources is limited (Hobfoll, 2011), the nurses’ presenteeism would result in a long-term consumption of their resources, which have not been supplemented and recovered, further causing an increase in fatigue, tension, and anxiety, so their work productivity is reduced (Sonnentag et al., 2012). More than that, nursing is also a job that takes a high emotional toll. When emotional resources have been excessively consumed, nurses may burn out and treat their patients in a negative manner (Demerouti et al., 2009). Consequently, it may be difficult for them to devote themselves to work, and their work energy may decrease as well.

Third, this study found that general self-efficacy played a moderation role between the presenteeism of nurses and productivity loss, which shows that general self-efficacy can effectively alleviate the negative impact of presenteeism on nurses’ productivity loss. These results support the opinion of Dew et al. (2005) that self-efficacy may play a role as a moderator in the relationship between presenteeism and its outcome variables. This could be because individuals with high general self-efficacy tend to adopt positive and problem-oriented coping strategies when faced with problems, while individuals with low general self-efficacy may tend to assume negative and emotional-coping strategies (Bandura, 1997). However, the former can effectively dissolve stress and keep those individuals healthier, both physically and mentally (Jex et al., 2001). Therefore, the nurses with high general self-efficacy, even in the face of high pressure and in poor physical condition, believe in themselves that they can accomplish their work tasks without producing negative emotions and feelings, such as tension, burnout, and frustration. Thus, they are able to maintain a high work performance. On the contrary, when adverse factors occur at work, nurses with low general self-efficacy often doubt their abilities, resulting in negative emotions, such as tension and anxiety, which eventually leads to lower work efficiency.

### Theoretical Implications

In recent years, scholars have attempted to generate a comprehensive and thorough understanding of presenteeism in the workplace. These findings extend the presenteeism literature in the following ways. First, this study clarified the distinction between presenteeism behavior and its outcome and investigated the relationship between them by introducing productivity loss as the outcome variable. In particular, these findings provided the relationship with robust empirical evidence. By analyzing the collected valid data from 340 nurses, the present study explicitly indicated that productivity loss is one of the detrimental consequences of presenteeism rather than a component of it. This research idea may provide a reference for future research. Furthermore, because most of the past studies were mainly conducted using European and American samples (Lin and Lu, 2013; Zhang and Li, 2016), this research selected Chinese nursing professionals as participants and operated in the Chinese working context. Therefore, to some extent, it fills the research gap by adding empirical data of a divergent population from a different cultural background.

Moreover, this research extends the presenteeism literature by building a new integrated framework. Based on the effort recovery theory (Meijman and Mulder, 1998) and the conservation of resources theory (Hobfoll, 2011), a comparatively complete analytical framework of presenteeism and its results was constructed by employing health as a mediator and general self-efficacy as the moderator. Precisely, results of the research confirmed that health plays a complete intermediary role between the relationship of nurses’ presenteeism and productivity loss and that general self-efficacy can buffer the negative effects of presenteeism on productivity loss. Despite this issue, the framework was confirmed with the empirical data, and all the research hypotheses were supported.

By using health as the mediation variables, this study reinforces the value of individuals’ physical and mental conditions on employees’ behaviors in job performance. Future researchers should examine other health-related variables to explore the acting mechanism of presenteeism in the work domain. Likewise, past studies rarely examined the roles of positive psychological factors in mitigating the undesirable outcomes from presenteeism. These findings highlight the importance of providing mental quality for the nursing professionals, who frequently work long shifts and work with illness. From this perspective, this study will not only enrich the empirical research of the domestic study of presenteeism and optimize the theories about presenteeism and its outcome variables but will further extend the research in the fields of occupational health psychology, epidemiology, public health, and other related areas.

### Practical Implications

This research has two main aspects of practical implications. First, from the perspective of medical and health management, as the present study confirmed, the nurses’ presenteeism causes a decline in their physical and mental health and their job performance and ultimately leads to productivity loss and increases organizations’ costs to cover it. Hence, we suggest that managers in the medical and health areas should be fully aware of the negative effects of presenteeism on their staff’s health and work performance, particularly in a society that attaches such importance to hard work and overtime work, for example, in the Chinese society. Although working with illness may help nurses to complete tasks to some extent, it also causes nurses to experience cumulative and incessant fatigue and stress, which will eventually result in a rise in the healthcare costs of organizations and the turnover of employees. Therefore, to reduce the negative impact of the nurse’s presenteeism on their productivity loss, the medical and health managers should initially pay attention to the prevention of nurses’ presenteeism, for example, establishing a multi-dimensional performance appraisal system, which highlights the quality of work rather than the frequency of attendance. Besides, medical and health organizations are advised to conduct tests or interviews to evaluate general self-efficacy as a reference for human resource recruitment and selection. Then, to reduce the work pressure in the nursing professions, it is necessary to build organizations with a supportive environment and the implementation of appropriate absence management. Meanwhile, establishing an effective mechanism of bumping procedures will reduce the frequency of nurses’ presenteeism as well, since it will allow nurses to recover from illness and stress, which will consequently facilitate work efficiency and further reduce organizations’ costs. Additionally, in an effort to diminish the detrimental effects of presenteeism on productivity, there is a strong demand for medical and health organizations to take actions to enhance nurses’ general self-efficacy in work, such as discovering and excavating nurses’ potential by providing them with training and professional development to improve their psychological quality and work-related abilities.

The second implication is from the perspective of the nurses themselves. To some extent, working despite having illness probably helps nurses to shape a personal image of being diligent and hardworking, which may promote their career development (Johns, 2010); however, the occurrence of presenteeism may eventually be counterproductive, as it will lead to the deterioration of the nurses’ health. Therefore, nurses should be aware of the jeopardy of presenteeism on their own health, work-related attitude and behavior, and their performance. It is advised that the nursing professions should take effective measures to reduce the frequency of presenteeism and its negative impact on them. At the same time, enhancing their expertise and skills in nursing could help them to improve their work efficiency and standard of care and allow them to provide better services for the rehabilitation and treatment of patients.

## Limitations and Future Research

However, there are three limitations of this research. The first limitation is about sampling. The participants of our study only include nurses from one hospital, and the valid sample size is as small as 340, so that the study fails to take full account of the differences between the work departments, such as internal medicine, surgery, emergency department, intensive care unit, etc. Moreover, some nurses have been working for a long time, which may lead to the survivorship bias; hence, it is not clear whether the results are universal among other hospitals or professional groups (such as enterprises and other public organizations). Compared with other occupations, due to heavy workload and low work substitutability, nurses tend to exhibit more presenteeism instead of absence in work (Bergstrom et al., 2009a,b). Additionally, all the participants are female; the male nurses’ presenteeism behavior may have its unique characteristics. Therefore, future research may expand the sample from various hospitals and examine the differences within the nurse population and could test our hypothetical models in different organizational contexts as well.

The second limitation focuses on the measurement of presenteeism behavior. The two-item Sickness Presenteeism Questionnaire (Lu et al., 2013) is a quick, reliable, and prevalently used measurement in the related theme of researches. However, with the increasing demand of acquiring deeper understanding on presenteeism and its associated variables, only a few questions and a few response options about the frequency of presenteeism may not able to identify differences sufficiently. Meanwhile, presenteeism is a sensitive phenomenon in workplaces that may trigger the social desirability of the participants in the survey. Future studies may consider developing systematic and multidimensional measurements with more items and by the use of other-evaluated assessments to prevent the occurrence of social desirability.

The third limitation is the method of cross-sectional study on the mechanism of nurse presenteeism and productivity loss. A cross-sectional study is a study of the characteristics of psychological and behavioral development in a short period of time. Hence, it is difficult to reflect the relationship between presenteeism and productivity loss systematically, comprehensively, and dynamically. Indeed, the results verified correlations among presenteeism, productivity loss, health, and general self-efficacy, the shortage that lies in the nature of the method determines it is difficult to capture the turning points in the functional relationship between the variables. In other word, it is not conducive to inferring the causal relationship between the variables. Therefore, in future research, a longitudinal design could be used to examine the interaction between the variables and whether the nurses’ presenteeism has a sustainable negative impact on productivity loss, so as to acquire a better understanding about the effects of nurses’ presenteeism.

## Conclusion

This study focused on the relationship between nurse’s presenteeism behavior and productivity loss. The results revealed that the nurse’s presenteeism behavior has a significant positive predictive effect on productivity loss, that is, the higher the frequency of presenteeism behavior, the greater the negative impact on nurses’ productivity. The results also confirmed the mediating role of health between nurse’s presenteeism behaviors and productivity loss and found that general self-efficacy could alleviate the negative effect of nurse’s presenteeism behaviors on productivity loss. The results of this study enrich the empirical research on presenteeism behavior, and improve the theoretical discussion on the behavior and its outcome variables, and further may expand the research on occupational health psychology, epidemiology, public health, and other fields.

In conclusion, presenteeism can significantly predict productivity loss in nurses, and hospital management should pay more efforts to strengthen the prevention and intervention of nurse presenteeism behavior. Moreover, it is important that future implementation efforts consider to improve the physical and mental health of nurses and enhance their self-efficacy level to reduce the negative impact of presenteeism on health-related productivity.

## Data Availability

The applications employed in this manuscript are freely available. Please contact the corresponding author for more details.

## Ethics Statement

The studies involving human participants were reviewed and approved by Henan University Institutional Review Board. The patients/participants provided their written informed consent to participate in this study.

## Author Contributions

YL is the principal investigator for the study, generated the idea and designed the study, was the primary writer of the manuscript, and approved all changes. JZ and SW supported the data input, data analysis and writing up the manuscript. SG supported the data collection. All authors were involved in developing, editing, reviewing, and providing feedback for this manuscript and have given approval of the final version to be published.

### Conflict of Interest Statement

The authors declare that the research was conducted in the absence of any commercial or financial relationships that could be construed as a potential conflict of interest.

## Abbreviations

GHQ-12: 12-Item general health questionnaire
GSES: General self-efficacy scale
SPQ: Sickness presenteeism questionnaire
SPS-6: Stanford presenteeism scale.

## References

[ref1] Aiken L. S. West S. G. (1991). Multiple regression: Testing and interpreting interactions. Newbury Park: Sage.

[ref2] Aronsson G. Gustafsson K. (2005). Sickness presenteeism: prevalence, attendance-pressure factors, and an outline of a model for research. J. Occup. Environ. Med. 47, 958–966. 10.1097/01.jom.0000177219.75677.17, PMID: 16155481

[ref3] Aronsson G. Gustafsson K. Dallner M. (2000). Sick but yet at work. An empirical study of sickness presenteeism. J. Epidemiol. Community Health 54, 502–509. 10.1136/jech.54.7.502, PMID: 10846192 PMC1731716

[ref4] Baker-McClearn D. Greasley K. Dale J. Griffith F. (2010). Absence management and presenteeism: the pressures on employees to attend work and the impact of attendance on performance. Hum. Resour. Manag. J. 20, 311–328. 10.1111/j.1748-8583.2009.00118.x

[ref5] Bandura A. (1997). Self-efficacy: The exercise of control. New York, US: Freeman.

[ref6] Beaton D. Bombardier C. Escorpizo R. Zhang W. Lacaille D. Boonen A.. (2009). Measuring worker productivity: frameworks and measures. J. Rheumatol. 36, 2100–2009. 10.3899/jrheum.090366, PMID: 19738221

[ref7] Bergstrom G. Bodin L. Hagberg J. Aronsson G. Josephson M. (2009a). Sickness presenteeism today, sickness absenteeism tomorrow? A prospective study on sickness presenteeism and future sickness absenteeism. J. Occup. Environ. Med. 51, 629–638. 10.1097/JOM.0b013e3181a8281b, PMID: 19448572

[ref8] Bergstrom G. Bodin L. Hagberg J. Lindh T. Aronsson G. Josephson M. (2009b). Does sickness presenteeism have an impact on future general health? Int. Arch. Occup. Environ. Health 82, 1179–1190. 10.1007/s00420-009-0433-6, PMID: 19504117

[ref9] Bierla I. Huver B. Richard S. (2013). New evidence on absenteeism and presenteeism. Int. J. Hum. Resour. Manag. 24, 1536–1550. 10.1080/09585192.2012.722120

[ref10] Biron C. Brun J. P. Ivers H. Cooper C. (2006). At work but ill: psychosocial work environment and well-being determinants of presenteeism propensity. J. Public Ment. Health 5, 26–37. 10.1108/17465729200600029

[ref11] Cai J. Qin H. Sun H. W. (2016). Stress, social support and mental health of clinical nurses. Chin. J. Health Psychol. 24, 189–193.

[ref12] Callahan D. (1973). “The WHO definition of ‘health’”. The Hastings Center Studies. 1, 77–87. 10.2307/3527467 4607284

[ref14] Demerouti E. Blanc P. M. L. Bakker A. B. Schaufeli W. B. Hox J. (2009). Present but sick: a three-wave study on job demands, presenteeism and burnout. Career Dev. Int. 14, 50–68. 10.1108/13620430910933574

[ref15] D’Errico A. Viotti S. Baratti A. Mottura B. Barocelli A. P. Tagna M.. (2013). Low back pain and associated presenteeism among hospital nursing staff. J. Occup. Health 55, 276–283. 10.1539/joh.12-0261-OA, PMID: 23796597

[ref16] Dew K. Keefe V. Small K. (2005). ‘Choosing’ to work when sick: workplace presenteeism. Soc. Sci. Med. 60, 2273–2282. 10.1016/j.socscimed.2004.10.022, PMID: 15748675

[ref17] Elstad J. I. Vabo M. (2008). Job stress, sickness absence and sickness presenteeism in nordic elderly care. Scand. J. Public Health 36, 467–474. 10.1177/1403494808089557, PMID: 18635730

[ref18] Feng D. D. Lu C. Q. Xiao A. L. (2008). Job insecurity, well-being, and job performance: the role of general self-efficacy. Acta Psychol. Sin. 40, 448–455. 10.3724/SP.J.1041.2008.00448

[ref19] Ferreira A. I. Martinez L. F. (2012). Presenteeism and burnout among teachers in public and private Portuguese elementary schools. Int. J. Hum. Resour. Manag. 23, 4380–4390. 10.1080/09585192.2012.667435

[ref20] Fryers T. Brugha T. Morgan Z. Smith J. Hill T. Carta M.. (2004). Prevalence of psychiatric disorder in Europe: the potential and reality of meta-analysis. Soc. Psychiatry Psychiatr. Epidemiol. 39, 899–905. 10.1007/s00127-004-0875-9, PMID: 15549242

[ref21] Gnambs T. Staufenbiel T. (2018). The structure of the General Health Questionnaire (GHQ-12): two meta-analytic factor analyses. Health Psychol. Rev. 12, 179–194. 10.1080/17437199.2018.1426484, PMID: 29325498

[ref22] Goetzel R. Z. Long S. R. Ozminkowski R. J. Hawkins K. Wang S. Lynch W. (2004). Health, absence, disability, and presenteeism cost estimates of certain physical and mental health conditions affecting US employers. J. Occup. Environ. Med. 46, 398–412. 10.1097/01.jom.0000121151.40413.bd, PMID: 15076658

[ref23] Goldberg D. P. (1972). The detection of psychiatric illness by questionnaire. London: Oxford University Press.

[ref24] Goldberg D. P. Gater R. Sartorius N. Ustun T. B. Piccinelli M. Gureje O. (1997). The validity of two versions of the GHQ in the WHO study of mental illness in general health care. Psychol. Med. 27, 191–197. 9122299 10.1017/s0033291796004242

[ref25] Gosselin E. Lemyre L. Corneil W. (2013). Presenteeism and absenteeism: differentiated understanding of related phenomena. J. Occup. Health Psychol. 18:75. 10.1037/a0030932, PMID: 23276197

[ref26] Gustafsson K. Marklund S. (2011). Consequences of sickness presence and sickness absence on health and work ability: a Swedish prospective cohort study. Int. J. Occup. Med. Environ. Health 24, 153–165. 10.2478/s13382-011-0013-3, PMID: 21526385

[ref27] Hansen C. D. Andersen J. H. (2008). Going ill to work-What personal circumstances, attitudes and work–related factors are associated with sickness presenteeism. Soc. Sci. Med. 67, 956–964. 10.1016/j.socscimed.2008.05.022, PMID: 18571821

[ref28] Hayes A. F. (2014). Introduction to mediation, moderation, and conditional process analysis: a regression-based approach. J. Educ. Meas. 51, 335–337. 10.1111/jedm.12050

[ref29] Hobfoll S. E. (2011). Conservation of resource caravans and engaged settings. J. Occup. Organ. Psychol. 84, 116–122. 10.1111/j.2044-8325.2010.02016.x

[ref30] Jex S. M. Bliese P. D. Buzzell S. Primeau J. (2001). The impact of self-efficacy on stressor–strain relations: coping style as an explanatory mechanism. J. Appl. Psychol. 86, 401–409. 10.1037/0021-9010.86.3.401, PMID: 11419800

[ref31] Johansson G. Lundberg I. (2004). Adjustment latitude and attendance requirements as determinants of sickness absence or attendance. Empirical tests of the illness flexibility model. Soc. Sci. Med. 58, 1857–1868. 10.1016/S0277-9536(03)00407-6, PMID: 15020004

[ref32] Johns G. (2010). Presenteeism in the workplace: a review and research agenda. J. Organ. Behav. 31, 519–542. 10.1002/job.630

[ref33] Johns G. (2011). Attendance dynamics at work: the antecedents and correlates of presenteeism, absenteeism, and productivity loss. J. Occup. Health Psychol. 16, 483–500. 10.1037/a0025153, PMID: 21875212

[ref34] Johns G. (2012). “Presenteeism: a short history and a cautionary tale” in Contemporary occupational health psychology: Global perspectives on research and practice. Vol. 2, eds. Houdmont J. Leka S. Siclair R. R. (Chichester, UK: Wiley-Blackwell), 204–220.

[ref35] Karimi L. Cheng C. Bartram T. Leggat S. G. Sarkeshik S. (2014). The effects of emotional intelligence and stress-related presenteeism on nurses’ well-being. Asia Pac. J. Hum. Resour. 53, 296–310. 10.1111/1744-7941.12049

[ref36] Kivimaki M. Head J. Ferrie J. E. Hemingway H. Shipley M. J. Vahtera J.. (2005). Working while ill as a risk factor for serious coronaryevents: the Whitehall II study. Am. J. Public Health 95, 98–102. 10.2105/AJPH.2003.035873, PMID: 15623867 PMC1449859

[ref37] Koopman C. Pelletier K. R. Murray J. F. Sharda C. E. Berger M. L. Turpin R. S.. (2002). Stanford presenteeism scale: health status and employee productivity. J. Occup. Environ. Med. 44, 14–20. 10.1097/00043764-200201000-00004, PMID: 11802460

[ref38] Koopmanschap D. M. Burdorf A. Jacob K. Meerding W. J. Brouwer W. Severens H. (2005). Measuring productivity changes in economic evaluation. PharmacoEconomics 23, 47–54. 10.2165/00019053-200523010-00004 15693727

[ref39] Letvak S. A. Ruhm C. J. Gupta S. N. (2012). Nurses’ presenteeism and its effects on self-reported quality of care and costs. Am. J. Nurs. 112, 30–38. 10.1097/01.NAJ.0000411176.15696.f9, PMID: 22261652

[ref41] Lin H. Y. Lu L. (2013). Presenteeism in workplace: constructing a cross-cultural framework. J. Hum. Resour. Manag. 13, 29–55.

[ref42] Lu L. Cooper C. L. Lin H. Y. (2013). A cross-cultural examination of presenteeism and supervisory support. Career Dev. Int. 18, 440–456. 10.1108/CDI-03-2013-0031

[ref43] Lu L. Peng S. Q. Lin H. Y. Cooper C. L. (2014). Presenteeism and health over time among Chinese employees: the moderating role of self-efficacy. Work Stress. 28, 165–178. 10.1080/02678373.2014.909904

[ref44] Lu C. Q. Siu O. L. Cooper C. L. (2005). Managers’ occupational stress in china: the role of self-efficacy. Personal. Individ. Differ. 38, 569–578. 10.1016/j.paid.2004.05.012

[ref45] McKevitt C. Morgan M. Dundas R. Holland W. W. (1997). Sickness absence and ‘working through’illness: a comparison of two professional groups. J. Public Health 19, 295–300. 10.1093/oxfordjournals.pubmed.a024633 9347453

[ref46] Meijman T. F. Mulder G. (1998). “Psychological aspects of workload” in A handbook of work and organizational psychology. 2nd Edn. Vol. 2, eds. Drenth P. J. D. Thierry H. de Wolff C. J. (Hove, UK: Psychology Press), 5–33.

[ref47] Pilette P. C. (2005). Presenteeism in nursing: a clear and present danger to productivity. J. Nurs. Adm. 35, 300–303. 10.1097/00005110-200506000-00006, PMID: 15951705

[ref49] Prasad M. Wahlqvist P. Shikiar R. Shih Y. C. T. (2004). A review of self-report instruments measuring health-related work productivity. PharmacoEconomics 22, 225–244. 10.2165/00019053-200422040-00002, PMID: 14974873

[ref50] Rantanen I. Tuominen R. (2011). Relative magnitude of presenteeism and absenteeism and work-related factors affecting them among health care professionals. Int. Arch. Occup. Environ. Health 84, 225–230. 10.1007/s00420-010-0604-5, PMID: 21140162

[ref51] Sasmita P. Sneha P. (2013). The determinants of sickness presenteeism. Indian J. Ind. Relat. 49, 256–269.

[ref52] Schwarzer R. Aristi B. (1997). Optimistic self-beliefs: assessment of general perceived self-efficacy in thirteen cultures. World Psychol. 3, 177–190.

[ref53] Sonnentag S. Mojza E. J. Demerouti E. Bakker A. B. (2012). Reciprocal relations between recovery and work engagement: the moderating role of job stressors. J. Appl. Psychol. 97, 842–853. 10.1037/a0028292, PMID: 22545619

[ref54] Sun J. M. Zhang Y. J. (2015). Presenteeism in the workplace: a new topic in organization and management research. Adv. Psychol. Sci. 23, 654–668. 10.3724/SP.J.1042.2015.00654

[ref55] Turpin R. S. Ozminkowski R. J. Sharda C. E. Collins J. J. Berger M. L. Billotti G. M.. (2004). Reliability and validity of the Stanford Presenteeism scale. J. Occup. Environ. Med. 46, 1123–1133. 10.1097/01.jom.0000144999.35675.a0, PMID: 15534499

[ref56] Virtanen M. Kivimaki M. Elovainio M. Vahtera J. Ferrie J. E. (2003). From insecure to secure employment: changes in work, health, health related behaviours, and sickness absence. Occup. Environ. Med. 60, 948–953. 10.1136/oem.60.12.948, PMID: 14634187 PMC1740437

[ref57] Wang C. K. Hu Z. F. Liu Y. (2001). Evidences for reliability and validity of the Chinese version of general self efficacy scale. Chin. J. Appl. Psychol. 7, 37–40.

[ref58] Weaver R. (2010). Cost of presenteeism surpasses absenteeism. Available at: http://www.examiner.com/human-capitalin-detroit/cost-of-presenteeism-surpasses-absenteeism (Accessed June 15, 2018).

[ref59] Zhang J. H. Guo S. J. Li Y. X. (2017). Measurement of nurses’ presenteeism and its related factors: a review. Chin. Ment. Health J. 31, 595–599.

[ref60] Zhang J. H. Li Y. X. (2016). Review on the act of presenteeism in the workplace. Psychol. Res. 9, 61–68.

[ref61] Zhao F. Dai J. M. Huang X. X. Fu H. (2011). Impacts of working population’s health on productivity: a quantitative analysis. J. Environ. Occup. Med. 28, 741–743.

[ref62] Zhao F. Dai J. M. Yan S. Y. Yang P. D. Fu H. (2010). Evidences for reliability and validity of the Chinese version of SPS-6. Chin. J. Ind. Hyg. Occup. Dis. 28, 679–682. 21126483

